# Quality of life in mucopolysaccharidoses: construction of a specific measure using the focus group technique

**DOI:** 10.1186/s13104-018-3157-4

**Published:** 2018-01-15

**Authors:** M. R. Oliveira, I. Schwartz, L. S. Costa, H. Maia, M. Ribeiro, L. B. Guerreiro, A. Acosta, N. S. Rocha

**Affiliations:** 1 Universidade Federal do Rio Grande do Sul, Hospital de Clinicas de Porto Alegre, Rua Ramiro Barcelos, 2350, Santa Cecília, Porto Alegre, RS 90035-903 Brazil; 2 Universidade Federal Fluminense, Rua Miguel de Frias, 9 Icaraí, Niterói, RJ 24220-008 Brazil; 3 Universidade Federal do Rio de Janeiro, Av. Pedro Calmon, 550, Cidade Universitária, Rio de Janeiro, RJ 21941-901 Brazil; 4 Universidade Federal da Bahia, R. Auristides, 2, Federacao, Salvador, BA 40210-340 Brazil; 5 Ramiro Barcelos, 2400 2º andar, Porto Alegre, RS 90 003-035 Brazil

**Keywords:** Mucopolysaccharidoses, Focus group, Scale development, Disease-specific QoL measure

## Abstract

**Objective:**

To describe the perceptions of patients, their caregivers, and their healthcare providers to the development of a new specific instrument for assessment of the quality of life (QoL) in patients with mucopolysaccharidoses (MPS) using a qualitative focus group (FG) design. FGs were held in two Brazilian states (Rio Grande do Sul and Rio de Janeiro).

**Results:**

Three versions of the new instrument were developed, each for a different age group: children (age 8–12 years), adolescents (age 13–17), and adults (age ≥ 18). The FGs mostly confirmed the relevance of items. All FGs unanimously agreed on the facets: School, Happiness, Life Prospects, Religiosity, Pain, Continuity of Treatment, Trust in Treatment, Relationship with Family, Relationship with Healthcare Providers, Acceptance, and Meaning of Life. The overall concept of QoL (as proposed by the WHO—World Health Organization) and its facets apply to this patient population. However, other specific facets—particularly concerning clinical manifestations and the reality of the disease—were suggested, confirming the need for the development of a specific QoL instrument for MPS.

**Electronic supplementary material:**

The online version of this article (10.1186/s13104-018-3157-4) contains supplementary material, which is available to authorized users.

## Introduction

The mucopolysaccharidoses (MPS) are rare genetic disorders of metabolism caused by the absence or malfunctioning of lysosomal enzymes [1–6]. Patients with MPS may have normal intelligence, but cognitive impairments are common. The broad range of clinical manifestations of MPS impacts on patient quality of life (QoL). Studies of Brazilian patients with different types of MPS have confirmed the severe morbidity associated with this group of disorders [7–12].

Assessment of QoL has been the object of increasing interest [13]. Several instruments for measurement of QoL have been studied in a variety of populations [14–19]; however, there are no specific instruments for assessment of QoL in patients with MPS. A review of the literature suggests that instruments for assessment of functional status correlate positively and significantly with QoL [20].

Given the importance of taking into account the viewpoints of patients and caregivers in the planning and assessment of health care [21], there is a lack of studies and appropriate instruments for QoL evaluation in patients with MPS. Hendriksz et al. [22] conducted a literature review to survey which QoL instruments that have been used to study MPS disorders. They concluded that many of these instruments have demonstrated that QoL is negatively impacted in patients with MPS. Overall, the most affected QoL domains were pain/discomfort [23] and mobility; problems with self-care or activities of daily living were also critical factors. Also, wheelchair use, unemployment, poor endurance, and poor pulmonary function were also associated with worse QoL [24–35]. The authors also noted the need for standardization and validation of QoL instruments for the different MPS disorders. This research gap suggests there is an unmet need for the development of a specific instrument for QoL assessment in patients with MPS. However, as these are very rare diseases, the construction of a specific QoL measure is particularly challenging because standard psychometric procedures (such as field testing using large samples) are impossible.

The ISPOR task force for newly developed patient-reported outcomes (PRO) instruments [36] recommended that, to ensure content validity, focus group (FGs) should be the initial qualitative step. The FG design is especially useful in the construction of culturally sensitive questionnaires or instruments for use in minority groups [37, 38].

Within this context, the present study describes the qualitative step of construction of a specific questionnaire for assessment of QoL in patients with MPS, with particular emphasis on the use of the FG method and its results.

## Main text

### Methods

This study is part of a larger project, composed of other stages, with the purpose of developing a specific instrument for assessment of QoL in patients with MPS. This project was designed in accordance with the methodology proposed by the WHOQOL group [19], and comprises the following stages: (1) review of the literature (originally from 1998 to 2008); (2) definition of the main facets relevant to an instrument for QoL in patients with MPS by a panel of Brazilian experts; (3) FG analysis of the expert-defined facets; (4) debriefing; (5) pilot module; (6) statistical analysis of pilot data; and (7) field testing. The present paper describes the qualitative step of FG technique (stage 3). The remaining four stages (4–7)—debriefing, administration of the pilot module, statistical analysis of pilot data, and field testing—will be described in other publications.

No descriptions of the final version of the questionnaire items will be provided in this paper; these will be the focus of a future article that will describe the field testing step.

#### Expert panel

Participants had been invited by e-mail. The panel comprised 15 healthcare providers that usually care for MPS patients (mainly clinical geneticists, pediatricians, and nurses), who proposed a 41-item instrument.

#### Focus group dynamics

Conceptually and characteristically, FG is a qualitative research method that involves a small number (ideally 5–10) of heterogeneous participants. The more heterogeneous the group is, the most diverse opinions and views will appear. The heterogeinity is one of the most important characteristics of this kind of qualitative methodology. The main purpose is to capture the opinions and viewpoints of the group (in our case, MPS patients).

Ten FGs were held following current standards for construction of PRO instruments [36]. This step took place in two Brazilian cities (Porto Alegre, state of Rio Grande do Sul, and Rio de Janeiro, state of Rio de Janeiro). Each group was meant to generate items for inclusion in the MPS QoL questionnaire, review and modify the facet definitions proposed by the expert panel, and propose additional aspects. The FGs were divided as follows: healthcare providers (HP) (group 1); caregivers or relatives of patients with MPS (group 2); children with MPS, between the ages of 8 and 12 (group 3); adolescents with MPS, between the ages of 13 and 17 (group 4); and adults (ADU) with MPS (group 5). Three versions of the instrument incorporating FG suggestions are Additional files 1, 2, 3.

All meetings were recorded and transcribed, and their content was assessed by two examiners: the lead author (MRO) and an investigator with experience in the FG technique (NSR) [39].

#### Subjects

Participant allocation across the FGs was carried out at the coordinating Center-Rio Grande do Sul (five FGs, one for each type of participant) and the Rio de Janeiro treatment center (also five FGs, one for each type of participant). Overall, 41 participants were included. Results are described in Table 1.

**Table 1:** Demographic profile of focus groups (n = 41)

	Healthcare providers	Caregivers and relatives of MPS patients	Adults with MPS	Adolescents with MPS	Children with MPS
Number of participants	14	10	6	3	8
State of origin	10 RS, 4 RJ	6 RS, 4 RJ	3 RS, 3 RJ	2 RS, 1 RJ	6 RS, 2 RJ
MPS type		I = 1 II = 5 III = 2 IVa = 1 VI = 1	I = 2 II = 3 VI = 1	IV = 2 VI = 1	II = 2 IVa = 2 VI = 4
Mean age	41	42.2	24	16	11
Range	(24–58)	(21–62)	(18–33)	(15–17)	(9–13)
Sex (%)					
Male/female	2 (14.3)/12 (85.7)	3 (30)/7 (70)	3 (50)/3 (50)	2 (66.6)/1 (33.3)	6 (75)**/**2 (25)
Employed/student (%)					
Yes	12 (100)	7 (70)	2 (33.3)	3 (100)	8 (100)
Duration of FG	3 h 20 min	2 h 40 min	2 h 15 min	1 h 10 min	1 h 15 min

*FG* focus group, *MPS* mucopolysaccharidoses, *RS* Rio Grande do Sul, *RJ* Rio de Janeiro

### Results

Among the proposed domains, the psychological domain engendered the most discussion to achieve saturation among all FGs.

#### Description of the focus group discussions

##### Physical domain

Activities of daily living: the HP FG believed it would be important to categorize these questions (with each item generating a different question) to facilitate understanding.

Functioning (will or motivation to do things): The HP group suggested that this facet include practical daily life activities, such as leisure, studies, etc. The caregiver and ADU groups suggested that the question is asked directly.

Energy (feeling lively or energetic): All FGs felt it would be important to keep this facet. Pain (usual pain): The caregiver and HP FGs believed this facet should measure pain intensity and frequency.

Sleep (sleep quality): The caregiver and HP FGs believed it would be important to assess sleep quality, as patients with MPS often experience airway obstruction, which leads to altered sleep.

Treatment (continuity and safety of treatment): All groups believed it would be important to define “treatment”.

##### Social domain

Family (activities and family relationship); Leisure (opportunities for and quality of leisure and recreation); School (satisfaction with school performance and relationships in school); Social relationships (quality of relationship with the community); Relationship with HP (quality of relationship with HP). All groups believed these facets should be kept.

##### Psychological domain

Autonomy (independence, freedom, and self-direction): All FGs, except for the children’s group, believed keeping this facet in the instrument was important.

Happiness (feeling cheerful), Dependence on parents (over protectiveness), Life prospects (plans for the future), Spirituality (influence of faith): All groups believed keeping these facets was very important.

Hope (regarding life): The ADU FG believed this facet should be excluded.

Death (thinking of death): The ADU FG and the caregiver FG diverged as to the relevance of this item. No consensus was achieved as to whether it should be kept or excluded.

##### Socio-economic domain

Social Security (retirement): The ADU FG suggested this facet should be excluded.

Finances (satisfaction with the financial situation), Work, Transportation (satisfaction with transportation), Knowledge of disease (information on the condition and quality of information on the condition): All groups believed these facets should be kept.

Rights (knowledge of one’s rights): The ADU FG suggested this facet should be excluded.

The originally proposed facets appear to have covered the most relevant aspects suggested (Table 2).

**Table 2:** Questions suggested by the adolescent MPS patient, adult MPS patient, children patient, caregiver, and healthcare providers focus groups for construction of the MPS QOL instrument, patient version

Focus group	Suggested question	Facet
Healthcare providers	Do you sleep well?	Sleep
Children with MPS	Do you wake up feeling tired or refreshed?	
Caregivers	How do you feel about your sleep?	
Healthcare providers	Do you get along well with your family?	Family
	Does your family provide support when you need it?	
	How is your family important in your life?	
Children with MPS	Do you love your family?	
	Are you treated well by your family?	
Healthcare providers	Do you get along well with your classmates?	School
Caregivers	Do you have enough freedom to do what you like?	Autonomy
Children with MPS	Do your parents do things for you (offer lots of help)?	
Caregivers	Are you satisfied with your life?	Satisfaction with life
Caregivers	Is having Social Security benefits important to you?	Social security
Healthcare providers	Would you need health insurance to supplement your treatment?	
Adults with MPS	Do you think your health condition gets in the way of your work?	Work
Healthcare providers	Do you think your rights are respected?	Rights
	Do you feel well-informed about your rights?	
Caregivers	Did you receive guidance about your condition?	Knowledge of disease
	How important is it to you to have knowledge of your condition?	
Healthcare providers	Do you have any doubts about your condition?	
	What is the extent of your knowledge (or understanding) about the hereditary nature of your condition?	
Healthcare providers	Are you satisfied with your care environment?	Treatment
Healthcare providers	Are you satisfied with your sex life?	Sex/love life
Healthcare providers	Are you satisfied with your love life?	
Children with MPS	Do you feel cheerful? Do you feel motivated to do things?	Energy
Children with MPS	Are you satisfied with your ability to play?	Leisure
Children with MPS	Do you feel like the others?	Appearance
Children with MPS	Do you feel that people ignore you?	Social relationships
Children with MPS	Are you satisfied with your savings? Insert $$	Finance

Among the various facets proposed, some received unanimous support across all groups (Table 3). Some of these were considered particularly important: Relationship with healthcare providers, Religiosity, Happiness, and Life prospects.

**Table 3:** Facets receiving unanimous support across all focus groups for construction of the MPS QOL instrument, patient version

Facet	Question
School	Are you satisfied with your school life?
	Do you get along well with your teacher?
Life prospects	Do you make plans for the future?
Religiosity	Do you believe faith can help you face your problems?
Pain	Do you experience pain?
Continuity of treatment	Are you concerned about the continuity of the care you receive?
Trust in treatment	Do you trust the treatment you receive?
Family	Can you go along with your family on activities (trips, birthdays, gatherings)?
Relationship with healthcare providers	Do you get along with the professionals who take care of your health?
Acceptance	Do you take the difficulties you face in stride?
Meaning of life	Do you think your life has meaning?

### Discussion

We are unaware of any studies that used the FG on patients with MPS. Furthermore, this was the first-Brazilian study to use the FG technique in children. This technique proved valid for assessment of the patient, caregiver, and HP perceptions of QoL in patients with MPS.

Overall, the facets proposed by the expert group were adequate and representative for development of an instrument to assess QoL in MPS.

Content analysis of these facets revealed that most were similar to the generic questions of the QoL assessment instrument, the WHOQOL-BREF, which covers the following aspects: overall QoL and general health perceptions; pain; dependence on medicinal substances; energy; sleep; mobility; work capacity; positive feelings; thinking, learning; self-esteem; bodily image; negative feelings, spirituality; personal relationships; sexual activity; home environment; financial resources; leisure; physical environment; and transport [19].

Facets that differed from those included in the WHOQOL instrument were associated with specific features of MPS. These included: treatment (continuity of treatment, access to treatment, trust in treatment, satisfaction); family (relationships, activities); leisure (play and sports); school (satisfaction with school life, difficulty keeping up, and relationship with teachers); relationship with HP; dependence on parents, religiosity; hope; death; affection; and knowledge of the disease.

Although MPS is a rare condition with a wide range of clinical presentations, the generic facets proposed by the WHOQOL group proved valid for this patient population [18]. However, this generic assessment did not cover the totality of elements necessary, as seen by the number of facets deemed worthy of inclusion that differed from the WHOQOL domains.

The testimonials represent the reflections and thoughts of those used to the reality of MPS. However, the viewpoints of HP on the QoL of patients with MPS stood out as somewhat different. In another study, caregivers (parents) tended to have a poorer perception of the health status of children with MPS than the children themselves, while HP’ assessments were more consistent with the perceptions reported by the children [40]. Our results were different, and this divergence may be explained by the differences among the types of MPS studied. Despite great diversity among participants and groups, there was unanimous agreement on several questions/aspects: School; Happiness; Life Prospects; Religiosity; Pain; Continuity of Treatment; Trust in Treatment; Relationship with Family; Relationship with HP; Acceptance; and Meaning of Life. However, the FGs also suggested that items could be added to the following facets: Happiness; Sleep; Family; Leisure; School; Stigmatization; Autonomy; Dependence on parents; Sex/Love Life; Social Security; Rights; Guidance/Knowledge of disease; and Work. Likewise, some FGs suggested that certain items be removed.

Particular attention should be given to the children’s group. Most Brazilian patients with MPS are children, and being able to give reliable information. Health assessment of children and adolescents, whether with generic or specific instruments, is a relevant research subject. Children as young as 5 can report reliably on concrete concepts such as pain and medication and, from the age of 9–10, can understand subjective concepts such as behavior and self-esteem [41]. Children over the age of 7 with asthma were assessed using a specific instrument and were able to understand the questions and their multiple-choice answers [41, 42].

### Conclusion

By using the FG technique, we were able to capture the perceptions of patients (including in children), caregivers, and HP about QoL in MPS. The overall concept of QoL and its facets, as proposed by the WHO, apply to this patient population. However, other specific facets, mostly related to the clinical manifestations of MPS and the reality of the disease, were proposed, thus confirming the need for the development of a specific QoL instrument for this group of disorders.

## Limitations

The small number of participants and inclusion restricted to patients with no mental illness might be regarded as a limitation. However, MPS is a rare group of genetic conditions, with no reliable estimates of its incidence in Brazil. Also, the next stages of development of the instrument will ascertain its ability to measure changes in health status or specific complains and to distinguish between disease stages and types, since the MPS are very heterogeneous in their clinical presentation.

## Additional files

**Additional file 1.** MPS QOL measure. Instrument for children.

**Additional file 2.** MPS QOL measure. Instrument for adolescents.

**Additional file 3.** MPS QOL measure, Instrument for adults.

## Abbreviations

QoL: quality of life
MPS: mucopolysaccharidoses
WHO: World Health Organization
FG: focus group
ISPOR: International Society for Pharmacoeconomics and Outcomes Research
PRO: patient-reported outcomes
WHOQOL group: World Health Organization quality of life group
HP: healthcare providers
ADU: adults
WHOQOL-BREF: World Health Organization quality of life instrument abbreviated version
