# Supplementary material for: Quality of life in mucopolysaccharidoses: construction of a specific measure using the focus group technique
Source: BMC Res Notes. 2018 Jan 15;11:28. doi: 10.1186/s13104-018-3157-4 (PMC5769464; doi:10.1186/s13104-018-3157-4)
Supplement: Supplementary file 1 — Additional file 1. MPS QOL measure. Instrument for children. [file 13104_2018_3157_MOESM1_ESM.doc]

**“Questionário 08 - 12”**

**Qualidade de Vida em MPS**

**Projeto Validação**

**Instruções**

Este questionário é sobre como você avalia sua qualidade de vida, saúde e outras áreas de sua vida. **Por favor, responda a todas as questões**. Se você não tem certeza sobre que resposta dar em uma questão, por favor, escolha entre as alternativas aquela que corresponda melhor à sua preferência, ou seja, aquela que você mais goste.

Por favor, tenha em mente que suas respostas têm como referência as **duas últimas semanas**.

Por favor, leia cada questão, veja o que você acha e circule no número que lhe parece a melhor resposta.

|  | ruim | mais ou menos | boa |
| --- | --- | --- | --- |
| 1. Você acha que sua vida é... | ☹ | 😐 | ☺ |
|  | 1 | 2 | 3 |

| ***Você está satisfeito com sua capacidade de:*** |  |  |  |
| --- | --- | --- | --- |
|  | pouco | mais ou menos | muito |
| 2. Tomar banho sozinho | ☹ | 😐 | ☺ |
|  | 1 | 2 | 3 |
| 3. Vestir-se sozinho | ☹ | 😐 | ☺ |
|  | 1 | 2 | 3 |
| 4. Alimentar-se sozinho | ☹ | 😐 | ☺ |
|  | 1 | 2 | 3 |
| 5. Caminhar sozinho | ☹ | 😐 | ☺ |
|  | 1 | 2 | 3 |
| 6. Locomover-se | ☹ | 😐 | ☺ |
|  | 1 | 2 | 3 |
| 7. Calçar-se sozinho | ☹ | 😐 | ☺ |
|  | 1 | 2 | 3 |
|  | sempre | às vezes | nunca |
| 8. Você se sente alegre | ☺ | 😐 | ☹ |
|  | 1 | 2 | 3 |
|  | sempre | às vezes | nunca |
| 9.Você se sente disposto (com energia) no seu dia-a-dia? | ☺ | 😐 | ☹ |
|  | 1 | 2 | 3 |

|  | sempre | às vezes | nunca |
| --- | --- | --- | --- |
| 10.Você se sente com vontade de fazer as coisas? | ☹ | 😐 | ☺ |
|  | 1 | 2 | 3 |

|  | muito | mais ou menos | pouco |
| --- | --- | --- | --- |
| 11.Você costuma ter dor no corpo? | ☹ | 😐 | ☺ |
|  | 1 | 2 | 3 |

|  | sempre | às vezes | nunca |
| --- | --- | --- | --- |
| 12.Você dorme cansado? | ☹ | 😐 | ☺ |
|  | 1 | 2 | 3 |

|  | nunca | às vezes | sempre |
| --- | --- | --- | --- |
| 13.Você dorme disposto? | ☹ | 😐 | ☺ |
|  | 1 | 2 | 3 |

|  | muito | mais ou menos | pouco |
| --- | --- | --- | --- |
| 14. O quanto o seu tratamento influencia no seu sono? | ☹ | 😐 | ☺ |
|  | 1 | 2 | 3 |

|  | muito | mais ou menos | pouco |
| --- | --- | --- | --- |
| 15. Você se sente feliz? | ☹ | 😐 | ☺ |
|  | 1 | 2 | 3 |

| **Tratamento** |  |  |  |
| --- | --- | --- | --- |
| ***As questões a seguir referem-se a todos os tratamentos que você realiza, incluindo consultas, terapias, exames, medicações e infusão.*** |  |  |  |
|  | pouco | mais ou menos | muito |
| 16. Você está satisfeito com o tratamento de saúde que voc~e recebe? | ☹ | 😐 | ☺ |
|  | 1 | 2 | 3 |

|  | nunca | às vezes | sempre |
| --- | --- | --- | --- |
| 17. Você consegue acompanhar as atividades da sua família? (passeios, aniversários, reuniões). | ☹ | 😐 | ☺ |
|  | 1 | 2 | 3 |

|  | pouco | mais ou menos | muito |
| --- | --- | --- | --- |
| 18.Quanta facilidade você tem para realizar suas atividades de lazer (por exemplo, brincar, jogar, andar de bicicleta, brincar com animais de estimação)? | ☹ | 😐 | ☺ |
|  | 1 | 2 | 3 |
| 19.Quanta facilidade você tem para praticar esportes? | ☹ | 😐 | ☺ |
|  | 1 | 2 | 3 |
|  | pouco | mais ou menos | muito |
| 20.Você está satisfeito com sua capacidade de brincar? | ☹ | 😐 | ☺ |
|  | 1 | 2 | 3 |
|  | muito | mais ou menos | pouco |
| 21.Você acha que a sua doença interfere nas suas brincadeiras? | ☹ | 😐 | ☺ |
|  | 1 | 2 | 3 |
|  | pouco | mais ou menos | muito |
| 22.Você está satisfeito com sua vida escolar? | ☹ | 😐 | ☺ |
|  | 1 | 2 | 3 |

|  | muito | mais ou menos | pouco |
| --- | --- | --- | --- |
| 23.Você acha difícil acompanhar seus estudos? | ☹ | 😐 | ☺ |
|  | 1 | 2 | 3 |

|  | pouco | mais ou menos | muito |
| --- | --- | --- | --- |
| 24.Você se dá bem com seu professor? | ☹ | 😐 | ☺ |
|  | 1 | 2 | 3 |
| 25.Você se sente igual aos outros? | ☹ | 😐 | ☺ |
|  | 1 | 2 | 3 |

|  | sempre | às vezes | nunca |
| --- | --- | --- | --- |
| 26.Você sente que as pessoas lhe ignoram? | ☹ | 😐 | ☺ |
|  | 1 | 2 | 3 |

|  | sempre | às vezes | nunca |
| --- | --- | --- | --- |
| 27.Você sente que as pessoas lhe rejeitam por seu problema de saúde? | ☹ | 😐 | ☺ |
|  | 1 | 2 | 3 |

|  | pouco | mais ou menos | muito |
| --- | --- | --- | --- |
| 28.Você aceita bem as dificuldades de sua vida? | ☹ | 😐 | ☺ |
|  | 1 | 2 | 3 |

|  | não | às vezes | sim |
| --- | --- | --- | --- |
| 29. Você acha que sua família tem dinheiro? | ☹ | 😐 | ☺ |
|  | 1 | 2 | 3 |

|  | pouco | | mais ou menos | muito |
| --- | --- | --- | --- | --- |
| 30.Você faz planos para o seu futuro? | ☹ | | 😐 | ☺ |
|  | 1 | | 2 | 3 |
| 31. Pensar na morte atrapalha a sua vida? | ☺ | 😐 | | ☹ |
|  | 1 | 2 | | 3 |

|  | pouco | mais ou menos | muito |
| --- | --- | --- | --- |
| 32. Quanto é importante para você ter conhecimento sobre sua doença? | ☹ | 😐 | ☺ |
|  | 1 | 2 | 3 |

|  | sim | mais ou menos | não |
| --- | --- | --- | --- |
| 33. Ter uma doença genética (hereditária) atrapalha sua vida? | ☹ | 😐 | ☺ |
|  | 1 | 2 | 3 |

Alguém lhe ajudou a preencher este questionário? ................................................

Quanto tempo você levou para preencher este questionário?...............................

Você tem algum comentário sobre o questionário?................................................

**OBRIGADO PELA SUA COLABORAÇÃO**
