# Supplementary material for: Quality of life in mucopolysaccharidoses: construction of a specific measure using the focus group technique
Source: BMC Res Notes. 2018 Jan 15;11:28. doi: 10.1186/s13104-018-3157-4 (PMC5769464; doi:10.1186/s13104-018-3157-4)
Supplement: Supplementary file 3 — Additional file 3. MPS QOL measure, Instrument for adults. [file 13104_2018_3157_MOESM3_ESM.doc]

**“Questionário acima 18”**

**Qualidade de Vida em MPS**

**Projeto validação**

**Instruções**

Este questionário é sobre como você avalia sua qualidade de vida, saúde e outras áreas de sua vida. **Por favor, responda a todas as questões**. Se você não tem certeza sobre que resposta dar em uma questão, por favor, escolha entre as alternativas aquela que corresponda melhor à sua preferência, ou seja, aquela que você mais goste.

Por favor, tenha em mente que suas respostas têm como referência as **duas** **últimas semanas**.

Por favor, leia cada questão, veja o que você acha e circule no número a que lhe parece a melhor resposta.

|  | pouco | mais ou menos | muita |
| --- | --- | --- | --- |
| 1.Você acha que a qualidade de sua vida é... | ☹ | 😐 | ☺ |
|  | 1 | 2 | 3 |
|  | ruim | mais ou menos | boa |
| 2.Você acha que sua saúde é... | ☹ | 😐 | ☺ |
|  | 1 | 2 | 3 |
| 3.Você acha que sua vida é... | ☹ | 😐 | ☺ |
|  | 1 | 2 | 3 |

| ***Você está satisfeito com sua capacidade de:*** |  |  |  |
| --- | --- | --- | --- |
|  | pouco | mais ou menos | muito |
| 4.Tomar banho sozinho | ☹ | 😐 | ☺ |
|  | 1 | 2 | 3 |
| 5. Vestir-se sozinho | ☹ | 😐 | ☺ |
|  | 1 | 2 | 3 |
| 6.Alimentar-se sozinho | ☹ | 😐 | ☺ |
|  | 1 | 2 | 3 |
| 7.Calçar-se sozinho | ☹ | 😐 | ☺ |
|  | 1 | 2 | 3 |
| 8.Caminhar sozinho | ☹ | 😐 | ☺ |
|  | 1 | 2 | 3 |
| 9.Locomover-se | ☹ | 😐 | ☺ |
|  | 1 | 2 | 3 |

|  | sempre | às vezes | nunca |
| --- | --- | --- | --- |
| 10.Você se sente disposto (com energia) no seu dia-a-dia? | ☺ | 😐 | ☹ |
|  | 1 | 2 | 3 |
| 11.Você costuma ter dor no corpo? | ☹ | 😐 | ☺ |
|  | 1 | 2 | 3 |

|  | ruim | mais ou menos | boa |
| --- | --- | --- | --- |
| 12.Como você avalia a qualidade do seu sono? | ☹ | 😐 | ☺ |
|  | 1 | 2 | 3 |

| **Tratamento** |  |  |  |
| --- | --- | --- | --- |
| ***Considerando todos os tratamentos que você faz, como: consultas, exames, medicamentos, terapias e infusão, responda as próximas 04 (quatro) perguntas.*** |  |  |  |
|  | pouco | mais ou menos | muito |
| 13.Você fica preocupado em não conseguir dar continuidade em seu tratamento de saúde? | ☺ | 😐 | ☹ |
|  | 1 | 2 | 3 |

|  | pouco | mais ou menos | muito |
| --- | --- | --- | --- |
| 14.Você confia no tratamento que você recebe? | ☹ | 😐 | ☺ |
|  | 1 | 2 | 3 |

|  | pouco | mais ou menos | muito |
| --- | --- | --- | --- |
| 15.Você está satisfeito com o relacionamento que você tem com seus familiares? | ☹ | 😐 | ☺ |
|  | 1 | 2 | 3 |
| 16.Você acha que precisa de sua família para levar a sua vida? | ☺ | 😐 | ☹ |
|  | 1 | 2 | 3 |

|  | pouco | mais ou menos | muito |
| --- | --- | --- | --- |
| 17.Quanta facilidade você tem para praticar esportes? | ☹ | 😐 | ☺ |
|  | 1 | 2 | 3 |

|  | pouco | mais ou menos | muito |
| --- | --- | --- | --- |
| 18.Você está satisfeito com seu lazer? | ☹ | 😐 | ☺ |
|  | 1 | 2 | 3 |

|  | pouco | mais ou menos | muito |
| --- | --- | --- | --- |
| 19. Você acha que a sua doença atrapalha suas atividades de lazer? | ☺ | 😐 | ☹ |
|  | 1 | 2 | 3 |

|  | pouco | mais ou menos | muito |
| --- | --- | --- | --- |
| 20. Você acha que sua doença interfere em seus estudos? | ☺ | 😐 | ☹ |
|  | 1 | 2 | 3 |

|  | pouco | mais ou menos | muito |
| --- | --- | --- | --- |
| 21. Você acha que tem independência suficiente para fazer o que deseja? | ☺ | 😐 | ☹ |
|  | 1 | 2 | 3 |

|  | nunca | às vezes | sempre |
| --- | --- | --- | --- |
| 22. Você sente que as pessoas lhe aceitam como você é? | ☹ | 😐 | ☺ |
|  | 1 | 2 | 3 |
| 23. Você se sente aceito como você é? | ☹ | 😐 | ☺ |
|  | 1 | 2 | 3 |
|  | não | às vezes | sim |
| 24. Você está satisfeito com a sua aparência? | ☹ | 😐 | ☺ |
|  | 1 | 2 | 3 |

|  | pouco | mais ou menos | muito |
| --- | --- | --- | --- |
| 25.Você se sente feliz? | ☹ | 😐 | ☺ |
|  | 1 | 2 | 3 |
| 26.Você acha que as pessoas que cuidam de você lhe protegem demais? | ☺ | 😐 | ☹ |
|  | 1 | 2 | 3 |
| 27.Você aceita as dificuldades de sua vida? | ☹ | 😐 | ☺ |
|  | 1 | 2 | 3 |

|  | pouco | mais ou menos | muito |
| --- | --- | --- | --- |
| 28.Você faz planos para o seu futuro? | ☹ | 😐 | ☺ |
|  | 1 | 2 | 3 |

|  | pouco | mais ou menos | muito |
| --- | --- | --- | --- |
| 29.Você sente que sua vida faz sentido? | ☹ | 😐 | ☺ |
|  | 1 | 2 | 3 |

|  | pouco | mais ou menos | muito |
| --- | --- | --- | --- |
| 30.Pensar na morte atrapalha a sua vida? | ☺ | 😐 | ☹ |
|  | 1 | 2 | 3 |

|  | pouco | mais ou menos | muito |
| --- | --- | --- | --- |
| 31.Você está satisfeito com sua vida amorosa? | ☹ | 😐 | ☺ |
|  | 1 | 2 | 3 |
| 32.Você está satisfeito com sua vida sexual? | ☹ | 😐 | ☺ |
|  | 1 | 2 | 3 |

|  | não | mais ou menos | sim |
| --- | --- | --- | --- |
| 33.Você acha que tem amigos suficientes? | ☹ | 😐 | ☺ |
|  | 1 | 2 | 3 |

|  | não | mais ou menos | sim |
| --- | --- | --- | --- |
| 34.Você está satisfeito com sua situação financeira? | ☹ | 😐 | ☺ |
|  | 1 | 2 | 3 |
| 35.Para você é importante ter o benefício do INSS? | ☹ | 😐 | ☺ |
|  | 1 | 2 | 3 |

|  | não | mais ou menos | sim |
| --- | --- | --- | --- |
| 36.Você se sente bem informado a respeito de seus direitos? | ☹ | 😐 | ☺ |
|  | 1 | 2 | 3 |
| 37.Você tem acesso aos meios de transporte de que necessita em sua vida? | ☹ | 😐 | ☺ |
|  | 1 | 2 | 3 |
|  | não | às vezes | sim |
| 38.Você acha que seu tratamento de saúde atrapalha seu trabalho? | ☺ | 😐 | ☹ |
|  | 1 | 2 | 3 |
| 39.Sua patologia interfere no desempenho do seu trabalho? | ☺ | 😐 | ☹ |
|  | 1 | 2 | 3 |
| 40.Você está satisfeito com sua vida profissional? | ☹ | 😐 | ☺ |
|  | 1 | 2 | 3 |

|  | pouco | mais ou menos | muito |
| --- | --- | --- | --- |
| 41.Você tem dúvidas com relação à sua doença? | ☺ | 😐 | ☹ |
|  | 1 | 2 | 3 |

|  | não | mais ou menos | sim |
| --- | --- | --- | --- |
| 42.Você foi orientado sobre a sua doença? | ☹ | 😐 | ☺ |
|  | 1 | 2 | 3 |

|  | nenhum | mais ou menos | muito |
| --- | --- | --- | --- |
| 43.Qual o grau de conhecimento (ou qual seu entendimento) sobre a hereditariedade da sua doença? | ☹ | 😐 | ☺ |
|  | 1 | 2 | 3 |

|  | não | mais ou menos | sim |
| --- | --- | --- | --- |
| 44.Ter uma doença genética (hereditária) atrapalha sua vida? | ☺ | 😐 | ☹ |
|  | 1 | 2 | 3 |

Alguém lhe ajudou a preencher este questionário? ..................................................

Quanto tempo você levou para preencher este questionário?...................................

Você tem algum comentário sobre o questionário?...................................................

**OBRIGADO PELA SUA COLABORAÇÃO**
